# The Potential of Isoprenoids in Adjuvant Cancer Therapy to Reduce Adverse Effects of Statins

**DOI:** 10.3389/fphar.2018.01515

**Published:** 2019-01-04

**Authors:** Huanbiao Mo, Rayna Jeter, Andrea Bachmann, Sophie T. Yount, Chwan-Li Shen, Hoda Yeganehjoo

**Affiliations:** ^1^Department of Nutrition, Byrdine F. Lewis College of Nursing and Health Professions, Georgia State University, Atlanta, GA, United States; ^2^Department of Clinical Nutrition, University of Texas Southwestern Medical Center, Dallas, TX, United States; ^3^Department of Chemistry, Georgia State University, Atlanta, GA, United States; ^4^Department of Pathology, Texas Tech University Health Sciences Center, Lubbock, TX, United States

**Keywords:** isoprenoids, HMG CoA reductase, mevalonate, SREBP, statin, synergy, cancer

## Abstract

The mevalonate pathway provides sterols for membrane structure and nonsterol intermediates for the post-translational modification and membrane anchorage of growth-related proteins, including the Ras, Rac, and Rho GTPase family. Mevalonate-derived products are also essential for the Hedgehog pathway, steroid hormone signaling, and the nuclear localization of Yes-associated protein and transcriptional co-activator with PDZ-binding motif, all of which playing roles in tumorigenesis and cancer stem cell function. The phosphatidylinositol-4,5-bisphosphate 3-kinase-AKT-mammalian target of rapamycin complex 1 pathway, p53 with gain-of-function mutation, and oncoprotein MYC upregulate the mevalonate pathway, whereas adenosine monophosphate-activated protein kinase and tumor suppressor protein RB are the downregulators. The rate-limiting enzyme, 3-hydroxy-3-methylglutaryl coenzyme A reductase (HMGCR), is under a multivalent regulation. Sterol regulatory element binding protein 2 mediates the sterol-controlled transcriptional downregulation of HMGCR. UbiA prenyltransferase domain-containing protein-1 regulates the ubiquitination and proteasome-mediated degradation of HMGCR, which is accelerated by 24, 25-dihydrolanosterol and the diterpene geranylgeraniol. Statins, competitive inhibitors of HMGCR, deplete cells of mevalonate-derived intermediates and consequently inhibit cell proliferation and induce apoptosis. Clinical application of statins is marred by dose-limiting toxicities and mixed outcomes on cancer risk, survival and mortality, partially resulting from the statin-mediated compensatory upregulation of HMGCR and indiscriminate inhibition of HMGCR in normal and tumor cells. Tumor HMGCR is resistant to the sterol-mediated transcriptional control; consequently, HMGCR is upregulated in cancers derived from adrenal gland, blood and lymph, brain, breast, colon, connective tissue, embryo, esophagus, liver, lung, ovary, pancreas, prostate, skin, and stomach. Nevertheless, tumor HMGCR remains sensitive to isoprenoid-mediated degradation. Isoprenoids including monoterpenes (carvacrol, L-carvone, geraniol, perillyl alcohol), sesquiterpenes (cacalol, farnesol, β-ionone), diterpene (geranylgeranyl acetone), “mixed” isoprenoids (tocotrienols), and their derivatives suppress the growth of tumor cells with little impact on non-malignant cells. In cancer cells derived from breast, colon, liver, mesothelium, prostate, pancreas, and skin, statins and isoprenoids, including tocotrienols, geraniol, limonene, β-ionone and perillyl alcohol, synergistically suppress cell proliferation and associated signaling pathways. A blend of dietary lovastatin and δ-tocotrienol, each at no-effect doses, suppress the growth of implanted murine B16 melanomas in C57BL6 mice. Isoprenoids have potential as adjuvant agents to reduce the toxicities of statins in cancer prevention or therapy.

## Introduction

Statins are competitive inhibitors of 3-hydroxy-3-methylglutaryl coenzyme A (HMG CoA) reductase (HMGCR), the rate-limiting enzyme in the mevalonate pathway (Goldstein and Brown, 1990). Widely prescribed as hypocholesterolemic agents, statins have been shown to inhibit cell proliferation and induce apoptosis in preclinical studies (Clendening and Penn, 2012). Clinical data suggest inverse association of statin use and risk of some, but not all, cancers. Dose-limiting toxicities of statins attributable to statin-induced compensatory upregulation of HMGCR and indiscriminate inhibition of HMGCR in normal and tumor cells pose constraints on the application of statins in cancer and call for novel approaches in reducing their side effects.

In this review we first summarize the role of the mevalonate pathway in cell proliferation and cancer by highlighting the intertwined relations between the mevalonate pathway and several key signaling molecules in growth regulation (Mullen et al., 2016). The multivalent regulation of HMGCR, including sterol-mediated transcriptional downregulation and nonsterol-induced enhancement of degradation, in normal cells contrasts with the sterol-resistant, dysregulated tumor HMGCR that remains uniquely sensitive to isoprenoid-mediated downregulation (Mo and Elson, 2004). The mechanisms underlying the isoprenoid-mediated tumor suppression is elucidated, followed by studies showing the synergistic effect of statins and isoprenoids and suggesting the potential of isoprenoids in adjuvant therapy to reduce the toxicities of statins.

## Role of the Mevalonate Pathway in Cell Proliferation and Cancer

**Figure 1:**
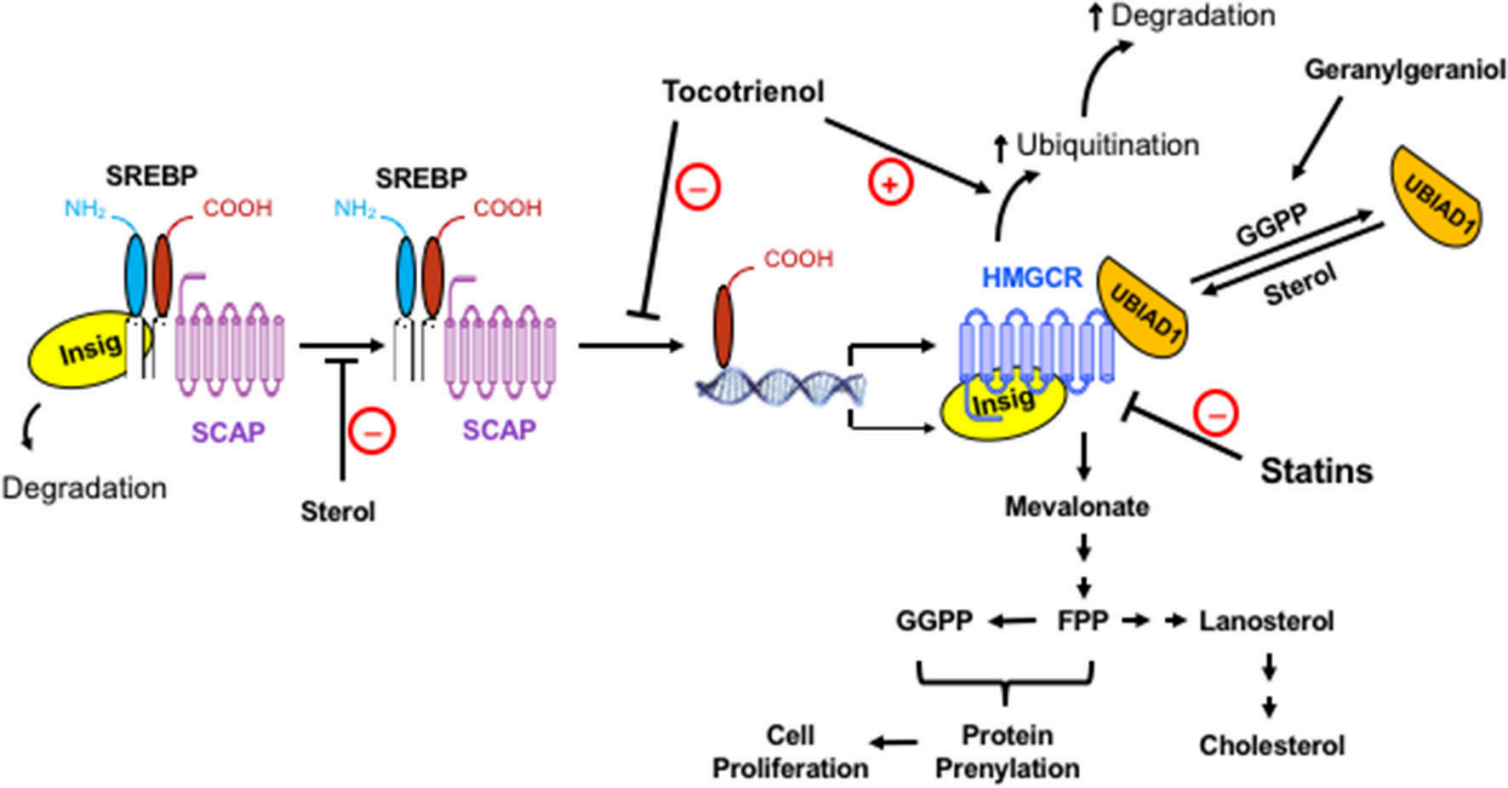
The regulation of the mevalonate pathway and the role of mevalonate-derived metabolites in cell proliferation. When cellular level of sterol is low, Insig dissociates from the SREBP-SCAP complex, allowing the latter to move to the Golgi apparatus. Following proteolytic cleavage of SREBP, its basic helix-loop-helix fragment enters the nucleus, binds to the SRE domain of target genes, and initiates the transcription and synthesis of a number of enzymes in the mevalonate pathway, including its rate-limiting enzyme, HMGCR. The activated mevalonate pathway produces sterols and nonsterols, including FPP and GGPP for protein prenylation and cell proliferation. Sterols block the transport of the SREBP-SCAP complex to the Golgi apparatus and induce the binding of HMGCR to Insig, leading to the ubiquitination and degradation of HMGCR. Exogenous geranylgeraniol is phosphorylated and converted to GGPP, which induces the dissociation of UBIAD1 from HMGCR and facilitates the degradation of HMGCR. The statins competitively inhibit HMGCR. Consequently, the lack of sterols and nonsterols results in a compensatory upregulation of HMGCR. Tocotrienols and potentially other isoprenoids block the processing and nuclear localization of SREBP and enhance the ubiquitination and degradation of HMGCR. HMGCR is upregulated in tumors but remains sensitive to isoprenoid-mediated downregulation. Synergistic impact of isoprenoids and statins on tumor HMGCR may offer a novel approach in enhancing the efficacy of statins with reduced toxicity.

The mevalonate pathway provides the bulk end product, cholesterol, and nonsterol isoprenoids such as heme-A, ubiquinone (or coenzyme Q_10_), dolichol, isopentenyl adenine, farnesyl pyrophosphate (FPP), and geranylgeranyl pyrophosphate (GGPP) (Goldstein and Brown, 1990). Cholesterol is important for membrane structure, whereas the mevalonate-derived nonsterol compounds play vital roles in iron-containing cofactors of hemoproteins (e.g., hemoglobin, myoglobin, catalase, endothelial nitric oxide synthase, and cytochrome), mitochondrial electron transport and cellular respiration, N-glycosylation of proteins, transfer RNA (Buhaescu and Izzedine, 2007), and post-translational prenylation and membrane anchorage of growth-related proteins, including the Ras, Rac, and Rho GTPase family (Hentschel et al., 2016; Wang and Casey, 2016). The prenylated proteins collectively support cell proliferation and cancer growth (Mullen et al., 2016) (Figure 1).

The mevalonate pathway is also intertwined with several signaling pathways with regulatory roles in cancer. The most frequently altered signaling pathway in cancer, the phosphatidylinositol-4,5-bisphosphate 3-kinase (PI3K)-AKT pathway, regulates cell survival and proliferation (Mullen et al., 2016). Through the downstream mammalian target of rapamycin (mTOR) complex 1 signaling (Duvel et al., 2010), PI3K-AKT pathway activates the mevalonate pathway (Ricoult et al., 2016) and concomitantly stimulates glucose uptake and glycolysis that provide acetyl-CoA and NADPH to support the mevalonate pathway. Gain-of-function mutations of p53 (Freed-Pastor et al., 2012), another frequently mutated gene in cancer, and the oncoprotein MYC (Wu et al., 2016), upregulate the transcription of mevalonate pathway genes. In contrast, adenosine monophosphate-activated protein kinase (AMPK), an energy sensor and central regulator of metabolism, downregulates the mevalonate pathway via phosphorylation (Beg et al., 1973, 1978) and transcriptional control (Li et al., 2011) of HMGCR. The tumor suppressor protein RB also downregulates the mevalonate pathway (Shamma et al., 2009). Conversely, the mevalonate pathway provides sterols and nonsterol products for Hedgehog pathway (Eaton, 2008), steroid hormone signaling (Nguyen et al., 2015), and the nuclear localization of Yes-associated protein (YAP) and transcriptional co-activator with PDZ-binding motif (TAZ) (Sorrentino et al., 2014; Koo and Guan, 2018), all of which have important roles in tumorigenesis and the cancer stem cell function (Mancini et al., 2018).

## Regulation of the Mevalonate Pathway in Normal Cells

In non-malignant cells, HMGCR is highly regulated by a multivalent feedback mechanism mediated by sterols and nonsterol isoprenoid end products of mevalonate metabolism. This tight regulatory feedback system, which operates at transcriptional and post-transcriptional levels, ensures proper signaling for sufficient sterol and isoprenoid syntheses and optimal cell growth (Goldstein and Brown, 1990). Transcription regulation, exerted by sterols and mediated by sterol regulatory element (SRE), is the dominant feedback mechanism. The SRE is a promoter sequence in the 5′ flanking region of more than 30 genes involved in lipid biosynthesis and uptake, including those of HMG CoA synthase, HMGCR, and low density lipoprotein (LDL) receptor (Goldstein and Brown, 1990; Brown and Goldstein, 1997; Horton et al., 2002; Goldstein et al., 2006). By providing a binding site for the membrane-bound transcriptional factors called SRE binding proteins (SREBPs), the SRE domain allows for SREBP-mediated transcriptional control of all these SRE-containing genes. Another ER membrane-embedded protein, known as SREBP cleavage activating protein (SCAP), is coupled with SREBP and plays a critical role in the intracellular escort and proteolytic processing of SREBP (Osborne et al., 1985; Gil et al., 1988; Goldstein et al., 2006). SCAP and HMGCR share an intramembrane sequence named sterol-sensing domain that detects the concentration of membrane and intracellular sterols and mediates the interactions of SCAP and HMGCR with insulin–induced gene (Insig) proteins during transcriptional and post-transcriptional regulations (Goldstein et al., 2006; Johnson and DeBose-Boyd, 2018).

When sterol levels diminish in cells, Insig dissociates from the SCAP-SREBP-Insig complex and undergoes ubiquitin-mediated degradation (Gong et al., 2006). The liberated SCAP-SREBP complex is then taken into vesicles coated by COPII proteins (Sec23, Sec24, and Sar1-GTP) and transported to the Golgi apparatus (Brown and Goldstein, 1999; Goldstein et al., 2006; Espenshade and Hughes, 2007). The SREBP is cleaved on the cytosolic surface of Golgi membrane in a two-step proteolytic reaction by site-1 serine protease and site-2 Zn^2+^ metalloproteinase, producing the transcriptionally active, basic helix-loop-helix fragment of SREBP. This active motif of SREBP enters nucleus, binds to the SRE domain, and initiates transcription of target genes that produce proteins taking part in cholesterol uptake and biosynthesis, including LDL receptor, HMG CoA synthase, HMGCR, Insig-1, and FPP synthase (Goldstein and Brown, 1990; Brown and Goldstein, 1999; Horton et al., 2002).

Once intracellular sterol levels are restored by increased biosynthesis or uptake due to aforementioned SREBP-upregulated events, a convergent feedback inhibition terminates the SREBP-directed transcriptional operation. Two of the SREBP-induced molecules, newly synthesized Insig-1 and newly biosynthesized or collected exogenous cholesterol, bind SCAP simultaneously and induce its conformational changes. As a result, the SCAP-SREBP complex is stabilized and retained in the ER membrane and prevented from undergoing any further transcriptional processing (Yang et al., 2002; Goldstein et al., 2006; Gong et al., 2006). This coordinated process ensures that cells attain sufficient amounts of both cholesterol and nonsterol isoprenoids for their growth and metabolic requirements before the SREBP transcriptional operation is fully halted. Furthermore, this feedback mechanism precludes overaccumulation of potentially toxic sterols.

As one of the genes in the mevalonate pathway under the transcriptional control of SREBP, HMGCR has a secondary line of regulation on its synthesis and degradation. While an earlier study postulated that an unknown mevalonate-derived isoprenoid regulates HMGCR translation via a 5′-untranslated region of HMGCR mRNA (Nakanishi et al., 1988), the mechanisms underlying the degradation of HMGCR have been more clearly elucidated. This post-transcriptional fine-tuning of HMGCR regulation is mediated by two mevalonate-derived compounds, a reduced tetracyclic triterpenoid, 24, 25-dihydrolanosterol, and a nonsterol diterpene, geranylgeraniol (Sever et al., 2003). 24, 25-Dihydrolanosterol and a less effective cholesterol induce binding of Insig to the sterol sensing domain of HMGCR, a process that requires a tetrapeptide sequence YIYF located in the second transmembrane segment of HMGCR (Roitelman and Simoni, 1992; McGee et al., 1996; Ravid et al., 2000; DeBose-Boyd, 2008). This binding is followed by the ubiquitination and proteasome-mediated degradation of HMGCR (Sever et al., 2004; Song et al., 2005; Lange et al., 2008; Nguyen et al., 2009). Addition of HMGCR inhibitors to cultured cells containing adequate amounts of sterols enhanced HMGCR protein expression and reduced its degradation, which was reverted with supplemental nonsterol metabolites (Goldstein and Brown, 1990), suggesting a nonsterol metabolite also regulates the expression and degradation of HMGCR. Using SV589 human fibroblasts that lack the monocarboxylate transporter and consequently the ability for mevalonate uptake, studies showed that geranylgeraniol is more potent than the 15-carbon isoprenoid farnesol in accelerating the sterol-induced and Insig-dependent ubiquitination and degradation of HMGCR (Sever et al., 2003).

More recent studies have shown that geranylgeraniol might be phosphorylated and converted to GGPP, which serves as a potent regulator of HMGCR stability and degradation (Garza et al., 2009; Schumacher et al., 2016, 2018). Buildup of GGPP in ER membranes triggers dissociation of UbiA prenyltransferase domain-containing protein-1 (UBIAD1) from HMGCR, permitting ER-to-Golgi transport and maximal degradation of HMGCR (Schumacher et al., 2018). Additionally, pulse-chase experiments confirmed the synergistic effects of mevalonate or geranylgeraniol, but not farnesol, with sterols in enhancing complete HMGCR degradation (Sever et al., 2003).

The E2 conjugating enzyme Ubc7, E3 ubiquitin ligase glycoprotein 78 (gp78), and ATPase VCP/p97 facilitate delivery of ubiquitinated HMGCR from the ER membrane to the proteasomes for proteolysis and degradation (Espenshade and Hughes, 2007). A recent study (Hwang et al., 2016) confirmed that liver employs the same mechanism regulating the accelerated degradation of HMGCR and cholesterol homeostasis initially found in cultured cells. This operation safeguards HMGCR synthesis and stability until the cellular requirements of sterols and isoprenoids are met (Goldstein and Brown, 1990; Peffley and Gayen, 2003; Sever et al., 2003; Song et al., 2005; Goldstein et al., 2006; Lange et al., 2008; Nguyen et al., 2009). It was recently reported that the monoterpene linalool prevents the binding of SREBP-2 to HMGCR promoter and enhances the ubiquitin-mediated degradation of HMGCR (Cho et al., 2011). A second monoterpene geraniol suppresses the protein level and specific activity of HMGCR in mouse liver (Galle et al., 2015) and A549 cells (Galle et al., 2014). These findings may add another layer of complexity to the regulation of HMGCR. It remains unknown whether GGPP mediates the effect of these monoterpenes on HMGCR.

## Upregulation of HMGCR in Tumors

Tumorigenesis has been associated with alterations and reprograming of energy, carbohydrate and lipid metabolisms with intertwined relations (Hanahan and Weinberg, 2011; Pavlova and Thompson, 2016). Activated oncogenes including *ras* and *myc*, for example, have been associated with alterations in glucose metabolism termed “aerobic glycolysis,” one of the hallmarks of cancer cells that is also known as the “Warburg effect” (Warburg, 1956; Hanahan and Weinberg, 2011). Hypoxia and the Ras oncoprotein that requires mevalonate-derived FPP for its post-translational modification can independently stimulate hypoxia-inducible factor 1α (HIF1α) and HIF2α transcriptional factors, which in turn upregulate glucose transporters and enzymes of the glycolytic pathway (Hanahan and Weinberg, 2011).

One of the metabolic adaptations to satisfy accelerated tumor growth is the upregulation of the mevalonate pathway, which has been implicated in the origin, progression, and phenotype of many human malignancies (Mo and Elson, 2004; Mullen et al., 2016). Tumor cells have augmented demands for nonsterol isoprenoids required for the prenylation of proteins supporting their excessive growth and proliferation. In response, tumors alter the monitory systems of mevalonate pathway that assure a constant and sufficient intracellular pool of sterols and nonsterol isoprenoids for non-malignant cell growth. One of the dysregulations stems from the overexpression and hyperactivity of tumor HMGCR as a result of its resistance to the sterol-dependent transcriptional regulation. Since Siperstein and Fagan (Siperstein and Fagan, 1964) reported the dysregulation of cholesterol synthesis in tumors and tumor-bearing animals, the uncoupling of HMGCR activity from sterol-mediated feedback regulation has been found in diverse tumors, embryonic and differentiating tissues and carcinogen-treated and regenerating liver (Elson et al., 1999).

Table 1 lists the studies showing differential expression of HMGCR in normal and tumor cells. In various human tissues including adrenal gland (Lehoux et al., 1984), blood and lymph (Yachnin and Mannickarottu, 1984; Harwood et al., 1991; Vitols et al., 1994; Hentosh et al., 2001; Kuzu et al., 2016), brain (Maltese, 1983; Laezza et al., 2015), breast (El-Sohemy and Archer, 2000; Celis et al., 2006; Ginestier et al., 2012), connective tissue (Kuzu et al., 2016), colon (Cerda et al., 1995; Hentosh et al., 2001; Notarnicola et al., 2004; Caruso and Notarnicola, 2005; Tyagi, 2005), embryo (Engstrom and Schofield, 1987), esophagus (Shi et al., 2013), liver (Kawata et al., 1990; Tam et al., 1991; Sohda et al., 2013), lung (Bennis et al., 1993), ovary (Zheng et al., 2018), prostate (Chen and Hughes-Fulford, 2001; Ettinger et al., 2004; Krycer et al., 2009; Murtola et al., 2012), skin (Kuzu et al., 2016), and stomach (Caruso et al., 2002), HMGCR mRNA and proteins levels are several-fold higher in tumors than their counterparts in normal tissues. Factors controlling the expression of HMGCR, including SREBP-1a, SREBP-2 and SCAP, were found to be overexpressed in prostate cancer (Krycer et al., 2009), contributing to transcriptional upregulation of HMGCR. Other enzymes in the mevalonate pathways, including farnesyltransferase, FPP synthase (Abate et al., 2017), and GGPP synthase (Wang et al., 2018), are also upregulated, suggesting a well-concerted regulation in raising the available metabolites derived from mevalonate. The augmented biosynthesis of cholesterol observed in tumors is consistent with the resistance of tumor HMGCR and SREBP-2 (Chen and Hughes-Fulford, 2001) to the cholesterol-mediated feedback inhibition that was initially observed in mouse hepatoma BW-7756 (Siperstein and Fagan, 1964). Several tissues in rodents, including bone marrow, breast (Rao et al., 1988; El-Sohemy and Archer, 2000), liver (Kandutsch and Hancock, 1971; Siperstein et al., 1971; Mitchell et al., 1978; Feingold et al., 1983; Gregg et al., 1986; Erickson et al., 1988; Azrolan and Coleman, 1989; Coni et al., 1992; Olsson et al., 1995; Clendening et al., 2010), lymph (Philippot et al., 1977), and pancreas (Rao et al., 1983), showed higher HMGCR expression and activity once they become malignant.

**Table 1:** Overexpression of HMGCR and upregulation of mevalonate pathway activities in tumors.

**Species**	**Tissues**	**Upregulation in tumors**	**References**
Human	Adrenal gland	Adrenal tumors had >70-fold higher HMGCR activity	Lehoux et al., 1984
	Blood and lymph	Mononuclear blood cells and leukocytes from leukemia patients had several-fold higher HMGCR activity than those from healthy subjects; HMGCR was upregulated by up to 50-fold in stimulated lymphocytes; upregulation of mevalonate pathway was correlated with lower survival of patients with acute myeloid leukemia	Yachnin and Mannickarottu, 1984; Harwood et al., 1991; Vitols et al., 1994; Hentosh et al., 2001; Kuzu et al., 2016
	Brain	HMGCR activity in metastatic brain tumors was higher than that in primary tumors; glioblastoma multiforme cell line U343 had higher HMGCR mRNA than normal human astrocytes; FPP synthase was overexpressed in glioblastoma compared to tumor-free peripheral brain and normal human astrocytes	Maltese, 1983; Laezza et al., 2015; Abate et al., 2017
	Breast	Upregulation of the mevalonate pathway enzymes including HMGCR was found in breast apocrine cysts, DMBA- and MNU- initiated mammary tumors, breast cancer stem cell-derived basal/mesenchymal tumorspheres, and Tamoxifen-resistant breast cancer	El-Sohemy and Archer, 2000; Celis et al., 2006; Ginestier et al., 2012
	Colon	HMGCR mRNA and activity were up to 8-fold higher in colorectal cancer cells than those in colon epithelial cells, fibroblasts and mucosa; farnesyltransferase and FPP synthase activities and cholesterol synthesis were also upregulated	Cerda et al., 1995; Hentosh et al., 2001; Notarnicola et al., 2004; Caruso and Notarnicola, 2005; Tyagi, 2005
	Connective tissue	Upregulation of mevalonate pathway was correlated with lower survival of sarcoma patients	Kuzu et al., 2016
	Embryo	HMGCR mRNA in embryonic tumors was higher than that in fetal tissues	Engstrom and Schofield, 1987
	Esophagus	HMGCR in esophageal squamous cell carcinoma was higher than that in esophageal tissue	Shi et al., 2013
	Liver	HMGCR activity and cholesterol biosynthesis were several-fold higher in hepatocellular carcinoma and human HepG2 and Hep3B hepatoma cells than those in liver, hepatocytes and fibroblasts	Kawata et al., 1990; Tam et al., 1991; Sohda et al., 2013
	Lung	HMGCR activity in A549 lung carcinoma cells was 2 to 4-fold higher than that in fibroblasts; overexpressed GGPP synthase was found in lung adenocarcinoma tissues and correlated with large tumors, high TNM stage, lymph node metastasis and poor prognosis	Bennis et al., 1993; Wang et al., 2018
	Ovary	SREBP-2 and HMGCR overexpression in several ovarian cancer cells including the cisplatin-resistant A2780 epithelial ovarian cancer cells	de Wolf et al., 2017; Zheng et al., 2018
	Prostate	PC-3, LNCaP, and VCaP prostate cancer cells had upregulated HMGCR, SREBP-2 and cholesterol biosynthesis in comparison to prostate epithelial cells and fibroblasts; following castration, LNCaP prostate tumor xenograft in athymic BALB/c nude mice progressed to androgen-independency with upregulated SREBP-1a,−1c, and−2, FPP synthase and SCAP in comparison to pre-castration LNCaP; SREBP-2 in human PrEC prostate epithelial cells and fibroblasts, but not that in DU145 or PC-3 prostate cancer cells, responds to 25-hydroxycholestgerol-mediated downregulation; patients with androgen-independent prostate cancer had higher SREBP-1	Chen and Hughes-Fulford, 2001; Ettinger et al., 2004; Krycer et al., 2009; Murtola et al., 2012
	Skin	Overexpression of HMGCR and other mevalonate pathway enzymes in melanoma; upregulation of mevalonate pathway was correlated with lower survival of melanoma patients	Kuzu et al., 2016
	Stomach	Gastric tumor had >2-fold increase in HMGCR	Caruso et al., 2002
Rat	Breast	DMBA- and MNU-induced mammary tumor had 2 to 4-fold higher HMGCR that was resistant to dietary cholesterol; total cholesterol in neoplastic tissue was 2 to 3-fold higher; neoplastic cholesterol synthesis was 5 to 6-fold higher	Rao et al., 1988; El-Sohemy and Archer, 2000
	Liver	HMGCR activities in hepatoma, carcinogen-induced hepatic nodules and preneoplastic foci were up to 14-fold higher than that in liver; HMGCR in hepatoma and preneoplastic foci was less responsive to cholesterol feedback	Siperstein et al., 1971; Mitchell et al., 1978; Feingold et al., 1983; Gregg et al., 1986; Erickson et al., 1988; Azrolan and Coleman, 1989; Coni et al., 1992; Olsson et al., 1995
	Pancreas	Tumor and fetal pancreas had higher HMGCR activity; fast-growing AT3A tumor had higher HMGCR activity than the slow-growing AT3B tumor	Rao et al., 1983
Mouse	Liver and bone marrow	Baseline HMGCR activity in liver tumors was 2 to 8-fold higher than that in liver; 1% dietary cholesterol led to >90% reduction in liver HMGCR but had much less response in hepatoma; ectopic expression of HMGCR in normal bone marrow or fetal liver cells increased myeloid colony formation	Kandutsch and Hancock, 1971; Clendening et al., 2010
Guinea pig	Lymph	L2C leukemic lymphocytes had >30 times higher cholesterol biosynthesis, 8 times higher HMGCR, and 25 times higher fatty acid biosynthesis than normal lymphocytes	Philippot et al., 1977

Consistent with these observations is the finding that exogenous mevalonate or artificially overexpressed HMGCR promotes growth. Supplemental mevalonate promoted growth of metastatic human breast cancer MDA-MB-435 cells in xenograft-bearing mice (Duncan et al., 2004a). Supplemental mevalonate increased the proliferation of cancer cells by upregulating cyclin-dependent kinase 2 (CDK2) activity and accelerating entry of cells into the S phase of cell division cycle (Duncan et al., 2004a). Ectopic expression of full-length or splice variant of HMGCR promotes transformation (Clendening et al., 2010). Finally, overexpression of mevalonate pathway genes is correlated with poor prognosis of recurrence-free and overall survival in breast cancer patients (Kimbung et al., 2016).

A recent study found that mutant p53-mediated upregulation of the mevalonate pathway is both necessary and sufficient for architectural phenotypes in breast cancer (Freed-Pastor et al., 2012), offering a new perspective for the role of the mevalonate pathway in cancer. The correlation between p53 mutation and highly expressed mevalonate pathway genes likely involves SREBP-2 and to a lesser extent, SREBP-1, which may assist the binding of p53 to the promoter of HMGCR gene. The mutant p53 and the mevalonate pathway also form a feed-forward loop in promoting cell proliferation. Mevalonate kinase upregulates DNAJ heat shock protein family (Hsp40) member A1 (DNAJA1), a type-I Hsp40, which inhibits the ubiquitination and degradation of mutant p53 mediated by the C-terminus of Hsc70-interacting protein (CHIP) E3 ubiquitin ligase (Parrales et al., 2016, 2018). Further implicating the role of the mevalonate pathway in tumorigenesis is the finding that mevalonate pathway inhibitors have cytostatic and cytotoxic effects in von Hippel-Lindau (VHL)-deficient clear cell renal cell carcinoma (CC-RCC) through an HIF-dependent mechanism (Thompson et al., 2018). Overall, these findings demonstrate the cancer promoting effects of excessive mevalonate and HMGCR activity.

## Effects of Statins on Cancer Growth With Dose-limiting Toxicities

Statins, competitive inhibitors of HMGCR and cholesterol-lowering drugs, possess anti-cancer properties due to their ability to suppress the mevalonate pathway (Clendening and Penn, 2012). Studies have suggested chemopreventive potential of statins against several types of cancer (Hindler et al., 2006) including those of blood, brain (Girgert et al., 1999; Koyuturk et al., 2004), bone (Kany et al., 2018), head and neck (Dimitroulakos et al., 2001; Knox et al., 2005), liver (Paragh et al., 2005), ovary (Abdullah et al., 2017; Jones et al., 2017), skin (Shellman et al., 2005), and thyroid (Chen et al., 2017). Statins deplete cells of mevalonate-derived isoprene metabolites (FPP and GGPP) that are essential for the prenylation and activation of oncoproteins, including Ras and Rho. Consequently, statins have been shown in preclinical *in vitro* and *in vivo* studies to modulate signaling molecules including H-, K-, and N-Ras, Raf-1, nuclear factor kappa B (NFκB), mitogen-activated protein kinases (MAPKs), PI3K/AKT, extracellular signal-regulated kinase (ERK), mTOR, signal transducer and activator of transcription 3 (STAT3), Janus kinase 2 (JAK2) and caspases, suppress cell proliferation and cell cycle progress, and induce tumor cell apoptosis (Hindler et al., 2006; Pisanti et al., 2014; Chen et al., 2015; Ahmadi et al., 2017; Beckwitt et al., 2018; Kong et al., 2018). Furthermore, statins inhibit tumor cell invasion, migration, and metastasis by attenuating the geranylgeranylation and activation of Rho oncoproteins (Al-Haidari et al., 2014; Kato et al., 2018). Conversely, mevalonate and GGPP abolished statin-induced effects on p-AKT, p-ERK, cell cycle arrest, and apoptosis in several tumors including human HL-60 leukemia cells (Chen et al., 2015), ovarian cancer cells (de Wolf et al., 2017), MiaPaCa-2 pancreatic cancer cells (Gbelcova et al., 2017), Caki-1 and KTC-26 renal carcinoma cells (Woschek et al., 2016), and malignant anaplastic thyroid cancer (Chen et al., 2017). By blocking the synthesis of mevalonate-derived metabolites that hinder the ubiquitination and degradation of mutant p53 protein, statins also suppress the growth of mutant p53-expressing cancer cells (Freed-Pastor et al., 2012; Freed-Pastor and Prives, 2016; Parrales et al., 2016). A recent study suggest that the anticancer effect of statins is associated with the epithelial-to-mesenchymal transition phenotype (Yu et al., 2018).

Clinical efficacy of statins in cancer reduction may be tissue specific. Statin use was found to be associated with lower risks of primary liver cancer (McGlynn et al., 2015), hepatocellular carcinoma (Kim et al., 2018), HPV-negative squamous cell carcinoma (SCC) of the larynx, hypopharynx, and nasopharynx (Lebo et al., 2018), and subtypes of non-Hodgkin lymphomas including diffuse large B-cell lymphomas and plasma cell lymphomas (Ye et al., 2018), reduced aggressiveness (Allott et al., 2016) and mortality (Yu et al., 2014) of prostate cancer, and lower cancer specific and all-cause mortalities in esophageal cancer (Nguyen et al., 2018). However, statins do not affect survival after colorectal cancer (Hoffmeister et al., 2015) and small-cell lung cancer (Seckl et al., 2017), the risk of pancreatic cancer (Hamada et al., 2018), or the progression of prostate cancer in certain minority-enriched subpopulations (Allott et al., 2018). The type and hydrophilicity of statins, length of statin use, and ethnicity, lifestyle, and preexisting health condition of subjects may have contributed to the diverse outcome in statin and cancer studies—with some but not all studies showing the anticancer effect of statins (Gong et al., 2017).

Reported dose-limiting toxicities of statins may further deter the use of statins—at least as single therapies—in cancer treatment. Observations in clinical practices note approximately 20% adverse reaction rates to statins (Bruckert et al., 2005; Maningat and Breslow, 2011; Zhang et al., 2013, 2017). Possible adverse effects include diabetes mellitus, hemorrhagic stroke, cognition decline, tendon rupture, interstitial lung disease, and muscle problems (Thompson et al., 2016). The Effect of Statins on Skeletal Muscle Function and Performance (STOMP) study suggested 5–10% myalgia incidence in statin users (Parker et al., 2013). Concerned about the potential adverse effects of statins on cognition, glycemic control, and incident diabetes as well as their wide range of interactions with other medications, the US Food and Drug Administration issued and implemented new safety labeling for statins in 2012.

The dose-limiting toxicities of statins may be at least partially attributed to statin-mediated compensatory upregulation of HMGCR. By mimicking and competing with HMG CoA, the substrate for HMGCR, statins render HMGCR unavailable for catalyzing the formation of mevalonate and downstream sterols and nonsterols including FPP and GGPP. Statins also change the conformation of HMGCR and prevent it from attaining a functional structure, attaining high efficacy and specificity in inhibiting HMGCR (Sirtori, 2014). By removing the sterol and nonsterol products that downregulate HMGCR, persistent exposure to statins induces compensatory overexpression of the HMGCR *in vitro* and *in vivo* (Roglans et al., 2002). Elevated HMGCR expression by approximately 6- to 10-folds has been observed in liver microsomes and fibroblasts (Bensch et al., 1978; Sirtori, 2014). In our hands lovastatin also induced upregulation of HMGCR in human DU145 prostate carcinoma cells (Yeganehjoo et al., 2017). Exposure to rosuvastatin, lovastatin, and atorvastatin caused 6-, 11-, and 15-fold greater expression of hepatic HMGCR protein, respectively, and enhanced SREBP-2 processing of some target genes in mice (Schonewille et al., 2016). Since majority of cholesterol synthesis occurs in the liver, the significant hepatic uptake of statins and the limited bioavailability of many statins in extra-hepatic tissues can cause compensatory induction of mevalonate pathway in these tissues. This can, in turn, stimulate overproduction of isoprenoid metabolites (FPP and GGPP) and downstream prenylated proteins (Duncan et al., 2005; Brown, 2007; Solomon and Freeman, 2008). A compensatory increase in HMGCR expression in extrahepatic tissues following cholesterol-lowering statin therapy may provide a mechanistic basis for the elevated risk of statin-related tumorigenesis observed in some studies (Sacks et al., 1996; Coogan et al., 2002; Shepherd et al., 2002; Beck et al., 2003).

In summary, preclinical and mechanistic studies support the anti-cancer property of statins. The indiscriminate inhibition of HMGCR in tumor and non-tumor cells and the resulted overexpression of HMGCR may have contributed to dose-limiting toxicities of statins and mixed outcomes in clinical investigations. Efforts have been devoted to identifying agents capable of synergizing with statins in cancer growth inhibition.

## Isoprenoids and Their Mechanisms of Action

Isoprenoids, also known as terpenoids, are a class of naturally occurring phytochemicals found in fruits, vegetables, and unrefined cereal grains. The carbon skeleton of these organic compounds is assembled from one or multiple copies of the five-carbon isoprene unit (X), giving rise to hemiterpenoids (1X, e.g., prenol and isovaleric acid), cyclic monoterpenes (2X, e.g., *d-*limonene, perillyl alcohol, perillaldehyde, carvacrol, carvone, and thymol), acyclic monoterpenes (2X, e.g., geraniol), sesquiterpenes (3X, e.g., cacalol, farnesol), diterpenes (4X, e.g., geranylgeraniol), triterpenes (6X, e.g., lupeol), tetraterpenes (8X, e.g., lycopene), and polyterpenes. Other isoprenoids such as the tocotrienols, members of the vitamin E family with a farnesyl side chain, are “mixed” isoprenoids with only part of the molecule being derived from the isoprene unit (Bach, 1995; Kumari et al., 2013). Besides the classical mevalonate pathway, an alternative non-mevalonate 2-C-methyl-D-erythritol 4-phosphate/1-deoxy-D-xylulose 5-phosphate (MEP/DOXP) pathway also produces isoprenoid precursors in bacteria, green algae, and some plants (Lichtenthaler, 1999; Hunter, 2007).

Diverse isoprenoids of plant mevalonate metabolism trigger post-transcriptional events to regulate the HMGCR expression and activity. These isoprenoid-mediated modulations include blockade of HMGCR mRNA translation and induction of HMGCR degradation in proteasomes.

Despite the demolished sterol-dependent transcriptional regulation in cancer cells, the isoprenoid-induced post-transcriptional tuning of HMGCR still exists and, in fact, becomes more compelling in tumor cells (Mo and Elson, 1999, 2004). Several isoprenoids such as δ-, γ-, and α-tocotrienol (Parker et al., 1993), farnesol (Meigs et al., 1996), β-ionone (Jones et al., 2013), geraniol (Houten et al., 2003; Peffley and Gayen, 2003), and their derivatives (Bradfute and Simoni, 1994; Pearce et al., 1994) suppress HMGCR activity. The 20-carbon isoprenoid geranylgeraniol effectively hinders HMGCR activity in human lung (Miquel et al., 1996) and prostate (Fernandes et al., 2013) tumor cells. At a concentration (30 μM) insufficient to affect SREBP-2 processing, geranylgeraniol synergized with sterols in shutting down processing of this HMGCR transcriptional factor in human SV589 fibroblasts (Sever et al., 2003). Tocotrienols inhibit mevalonate pathway through an Insig-dependent ubiquitination and degradation of HMGCR (Song and DeBose-Boyd, 2006). This regulation of HMGCR by tocotrienols has endowed these molecules with regulatory effects on cholesterol production and, thus, their hypocholesterolemic properties in mammalian cells (Parker et al., 1993). δ- and γ-tocotrienols, the strongest suppressors of HMGCR (Pearce et al., 1994; Song and DeBose-Boyd, 2006), were initially found to mimic nonsterol isoprenoids in accelerating HMGCR degradation at the post-transcriptional level (Parker et al., 1993). Subsequent studies revealed the direct recognition of these two structures by sterol-sensing systems in the ER membrane and their sterol-like activities in enhancing the Insig-dependent ubiquitination and degradation of HMGCR (Song and DeBose-Boyd, 2006). The same study delineated efficient blocking of SREBP-2 processing by the δ- but not γ- isoform of tocotrienol (Song and DeBose-Boyd, 2006). Additionally, combination of mevalonate or a mevalonate-derived nonsterol product (e.g., geranylgeraniol) with either δ- or γ-tocotrienol maximally eliminated HMGCR expression in these experiments (Song and DeBose-Boyd, 2006). A later study found tocotrienols suppress SREBP-2 activity by degrading mature SREBP-2 in the nucleus via a proteasome-independent mechanism in a human LNCaP-364 androgen-independent prostate cancer cell line (Krycer et al., 2012). Consequently, tocotrienols downregulate the HMGCR mRNA level.

By depleting tumor cells of mevalonate-derived intermediates, such as FPP and GGPP, isoprenoids can interrupt the prenylation and modification of critical signaling proteins including Ras, lamin B, and other growth related proteins and, thus, suppress tumor growth and proliferation (Mo and Elson, 1999, 2004; Yeganehjoo et al., 2017). Numerous studies have been conducted to unveil the significant outcomes of isoprenoid-based manipulations of the mevalonate pathway and HMGCR in cancer cells. γ-Tocotrienol at 0–30 μM downregulated HMGCR, membrane H-, K-, and N-Ras, and Raf-1, p-AKT, and p-ERK in HL-60 cells (Chen et al., 2015), adding to a long list of *in vitro* and *in vivo* studies showing isoprenoid-mediated suppression of the growth of cancer cells including those of blood (Shoff et al., 1991; Melnykovych et al., 1992; Mo and Elson, 1999; Lee et al., 2015), breast (Iqbal et al., 2004; Pierpaoli et al., 2010, 2013; Gomide et al., 2013; Ding et al., 2017), cervix (Yazlovitskaya and Melnykovych, 1995; Xu et al., 2017; Potocnjak et al., 2018), colon (Mo and Elson, 1999; Gomide et al., 2013, 2016), liver (Wada et al., 2005; Crespo et al., 2013; Scolastici et al., 2014; Rodenak-Kladniew et al., 2018), lung (Mo and Elson, 1999; Wada et al., 2005; Gomide et al., 2013; Galle et al., 2014), mouth (Liang et al., 2013; Madankumar et al., 2013), pancreas (Hussein and Mo, 2009; Fernandes et al., 2013), prostate (Mo and Elson, 2004; Sundin et al., 2012; Fernandes et al., 2013; Jones et al., 2013; Yeganehjoo et al., 2017), skin (Mo et al., 2000; McAnally et al., 2007; Chang et al., 2009; Chaudhary et al., 2013), and stomach (Dong et al., 2013; Liu et al., 2013). Concomitantly, isoprenoids induce cell cycle arrest and apoptosis in the tumor cells (Mo and Elson, 1999, 2004; Dong et al., 2013; Jones et al., 2013; Yeganehjoo et al., 2017; Rodenak-Kladniew et al., 2018; Sailo et al., 2018).

More importantly, differing from the statin-mediated indiscriminate inhibition of HMGCR in normal and tumor cells, the differential responses of HMGCR to isoprenoid-mediated downregulation render tumor cells more sensitive to isoprenoid-induced growth suppression (Mo et al., 2013). Non-tumor cells including aortic epithelial cells (Adany et al., 1994), fibroblast (Yazlovitskaya and Melnykovych, 1995; Ura et al., 1998; Mo and Elson, 1999; Smalley and Eisen, 2002; Yano et al., 2005), primary hemopoietic cells (Rioja et al., 2000), hepatocytes (Pearce et al., 1992; Ruch and Sigler, 1994; Sakai et al., 2004; Har and Keong, 2005; Yin et al., 2012), mammary epithelial cells (McIntyre et al., 2000; Duncan et al., 2004b; Shun et al., 2004; Yap et al., 2010; Liu et al., 2011; Patel and Thakkar, 2014), myeloid cells (Sahin et al., 1999), pancreatic ductal epithelial cells (Stayrook et al., 1997), prostate cells (Adany et al., 1994; Srivastava and Gupta, 2006), and umbilical vein endothelial cells (HUVEC) (Yoshikawa et al., 2010), are much less susceptible to the isoprenoid-mediated growth inhibition, suggesting that isoprenoids exert tumor-targeted growth inhibitory effects (Elson et al., 1999; Mo and Elson, 2004). Isoprenoid-rich extracts such as tocotrienol-rich fraction (TRF) of palm oil selectively and significantly inhibit growth and induce apoptosis of human prostate cancer LNCaP, DU145, and PC-3 cells with no significant impact on the viability of normal human prostate epithelial cells (Srivastava and Gupta, 2006). Similarly, tocotrienol stimulate growth inhibition, caspase activity, DNA fragmentation, and apoptosis in rat hepatoma dRLh-84 cells without affecting normal RLN-10 hepatocytes (Sakai et al., 2004). Tocotrienols also induce apoptosis of estrogen-non-responsive MDA-MB-435 and estrogen-responsive MCF-7 human breast cancer cells with no or lower levels of impact in normal human mammary epithelial cells and immortalized but non-tumorigenic human MCF-10A cells (McIntyre et al., 2000; Shun et al., 2004; Yap et al., 2010). As summarized in Table 2, isoprenoids such as monoterpenes (carvacrol, L-carvone, geraniol, perillyl alcohol), sesquiterpenes (cacalol, farnesol, β-ionone), diterpene (geranylgeranyl acetone), and “mixed” isoprenoids (tocotrienols) and their derivatives exert tumor-targeted growth inhibition with no-to-minimal impacts on the viability and morphology of normal healthy cells.

**Table 2:** Differential sensitivities of tumor and non-tumor cells to isoprenoids-mediated HMGCR downregulation, growth suppression and apoptosis.

**Isoprenoids**	**Cell lines in comparison**	**Differential impacts**	**References**
Carvacrol	Human hepatocellular carcinoma HepG2 cells vs. LO2 hepatocytes	Carvacrol (0–0.4 mM) suppressed HepG2 cell survival with no impact on LO2 cells	Yin et al., 2012
L-Carvone	MCF7 and MDA MB-231 vs. MCF10A	IC50^*^ for MCF10A (20 mM) was 20-fold higher than that those for MCF7 and MDA MB-231 (1 mM)	Patel and Thakkar, 2014
Geraniol	Human MCF-7 mammary tumor cells vs. MCF-10F normal breast epithelial cells	Higher impact on MCF-7 cell growth	Duncan et al., 2004b
Perillyl alcohol	Viral Ha-*ras*- and *raf*-transformed rat liver epithelial cells WB-*ras* (200 μM) and R3611-3 (250 μM) vs. non-transformed rat liver epithelial cell WB-neo and RLEC-2	IC50 for epithelial cells (400 μM) was higher than those for transformed cells (200–250 μM)	Ruch and Sigler, 1994
	Hamster B12/13 pancreatic ductal adenocarcinoma cells vs. D27 pancreatic ductal epithelial cells	IC50 for D27 cells (270 μM) nearly doubled that for B12/13 cells (150 μM); apoptosis and BAK expression in B12/13 cells	Stayrook et al., 1997
	Murine Bcr/Abl-transformed FDC.P1 and 32D myeloid cells vs. non-transformed myeloid cells	IC50 values for non-transformed cells were much higher than those for transformed cells	Sahin et al., 1999
Cacalol	Human MCF7 and MDA-MB231 mammary carcinoma cells vs. MCF10A and HBL-100 epithelial cells	Cacalol (35 and 70 μM) induced apoptosis in tumor cells but not epithelial cells	Liu et al., 2011
Farnesol and derivatives	Human HeLa-S3k and C-4-1 cervical carcinoma cells vs. human CF-3 newborn foreskin fibroblasts and porcine aortic endothelial cells (PAC); Mouse L5178Y-R (tumorigenic) lymphoma cells vs. L5178Y-S (non-tumorigenic) cells; Human DU145 prostate carcinoma cells vs. normal prostate cells	Tumor cells were several-fold more sensitive than non-tumor cells to farnesol-mediated growth inhibition	Adany et al., 1994; Yazlovitskaya and Melnykovych, 1995
	Ki-ras-transformed fibroblasts vs. NIH 3T3	IC50 for farnesylamine was 20-fold higher in NIH 3T3 cells	Ura et al., 1998
	Leukemia vs. human primary hemopoietic cells	Farnesol induced apoptosis in leukemic blasts from acute myeloid leukemia patients, but not in primary T-lymphocytes;	Rioja et al., 2000
	Murine B16 melanoma cells vs. NIH 3T3 fibroblasts	Farnesylthiosalicylic acid inhibited the growth of B16 melanoma cells with no impact on NIH 3T3 fibroblasts	Smalley and Eisen, 2002
β-Ionone	Human Caco2 colon adenocarcinoma cells vs. CCD-18 Co fibroblasts	IC50 was 3-fold higher in fibroblasts	Mo and Elson, 1999
	Human MCF-7 mammary tumor cells vs. MCF-10F normal breast epithelial cells	β-Ionone (500 μM) inhibited MCF-7 and MCF-10F cell growth by 80 and 38%, respectively	Duncan et al., 2004b
Geranylgeranyl-acetone (GGA)	Human DLD-1 and HT29 colon cancer cells vs. umbilical vein endothelial cells (HUVEC)	GGA (50 and 100 μM) suppressed the proliferation of DLD-1 and HT29 with no impact on that of HUVEC	Yoshikawa et al., 2010
Tocotrienols and derivatives	Human HepG2 hepatoma cells vs. primary rat hepatocytes	IC50 values were ~ 100-fold higher in hepatocytes in inhibiting acetate incorporation into digitonin-precipitable sterols	Pearce et al., 1992
	Highly malignant mammary tumor cells vs. preneoplastic mouse mammary epithelial cells; MCF-7, MDA-MB231, and MDA-MB-435 mammary tumor cells vs. MCF10A mammary epithelia cells	IC50 values were higher for epithelial and preneoplastic cells; tumor cells had higher apoptotic response	McIntyre et al., 2000; Shun et al., 2004; Yap et al., 2010
	Human A549 lung carcinoma cells vs. NIH 3T3 fibroblasts	A549 more sensitive to 6-O-carboxypropyl-a-tocotrienol	Yano et al., 2005
	Human LNCaP, PC-3 and DU145 prostate tumor cells vs. human PZ-HPV-7 virally transformed normal prostate epithelial cells	IC50 values for tocotrienol-rich fraction were 3 to 5-fold higher in normal cells; induced apoptosis in tumor but not normal cells	Srivastava and Gupta, 2006
	Rat dRLh-84 hepatoma cells vs. RLN-10 hepatocytes	Higher impact on cell viability, caspase activation, and apoptosis in tumor cells	Sakai et al., 2004
	Murine BNL 1ME A.7R.1 liver cancer cells vs. BNL CL.2 normal liver cells	Higher impact on cell viability, caspase-3 activation and DNA fragmentation in tumor cells	Har and Keong, 2005

* IC50: concentrations of compounds required to inhibit cell proliferation by 50%

The isoprenoid-mediated degradation of HMGCR complements the sterol-mediated transcriptional regulation in normal cells. In tumor cells with HMGCR that is resistant to sterol feedback, post-transcriptional downregulation of HMGCR by isoprenoids leads to suppression of cell proliferation and induction of apoptosis; concomitantly isoprenoids have minimal impact on normal cells. This unique tumor-targeting property of isoprenoids affords them potential in adjuvant therapy with statins.

## Synergistic Effect of Statins and Isoprenoids on Tumor Growth may Reduce Statin Toxicity

Since long-term use of a single compound requires a relatively high dose, which tends to be associated with adverse effects, synergistic effects of therapeutic compounds can provide the same or enhanced efficacy with lower doses of each agent and plausibly fewer adverse effects to lesser degrees. Investigations with blends of isoprenoids have shown favorable outcomes. Studies using tumor cells of skin (Mo and Elson, 1999; McAnally et al., 2007), prostate (Mo and Elson, 2004), and pancreas (Hussein and Mo, 2009) have delineated the synergistic or cumulative effects of HMGCR suppressors on mevalonate signaling and tumor cell growth. Geranylgeraniol and *d*-δ-tocotrienol synergistically suppressed growth of the murine b16 melanoma cells (Katuru et al., 2011). Dietary γ-tocotrienol (2 mmol/kg) and β-ionone (2 mmol/kg) administered individually or together also improved survival in mice bearing implanted melanomas (He et al., 1997).

**Figure 2:**
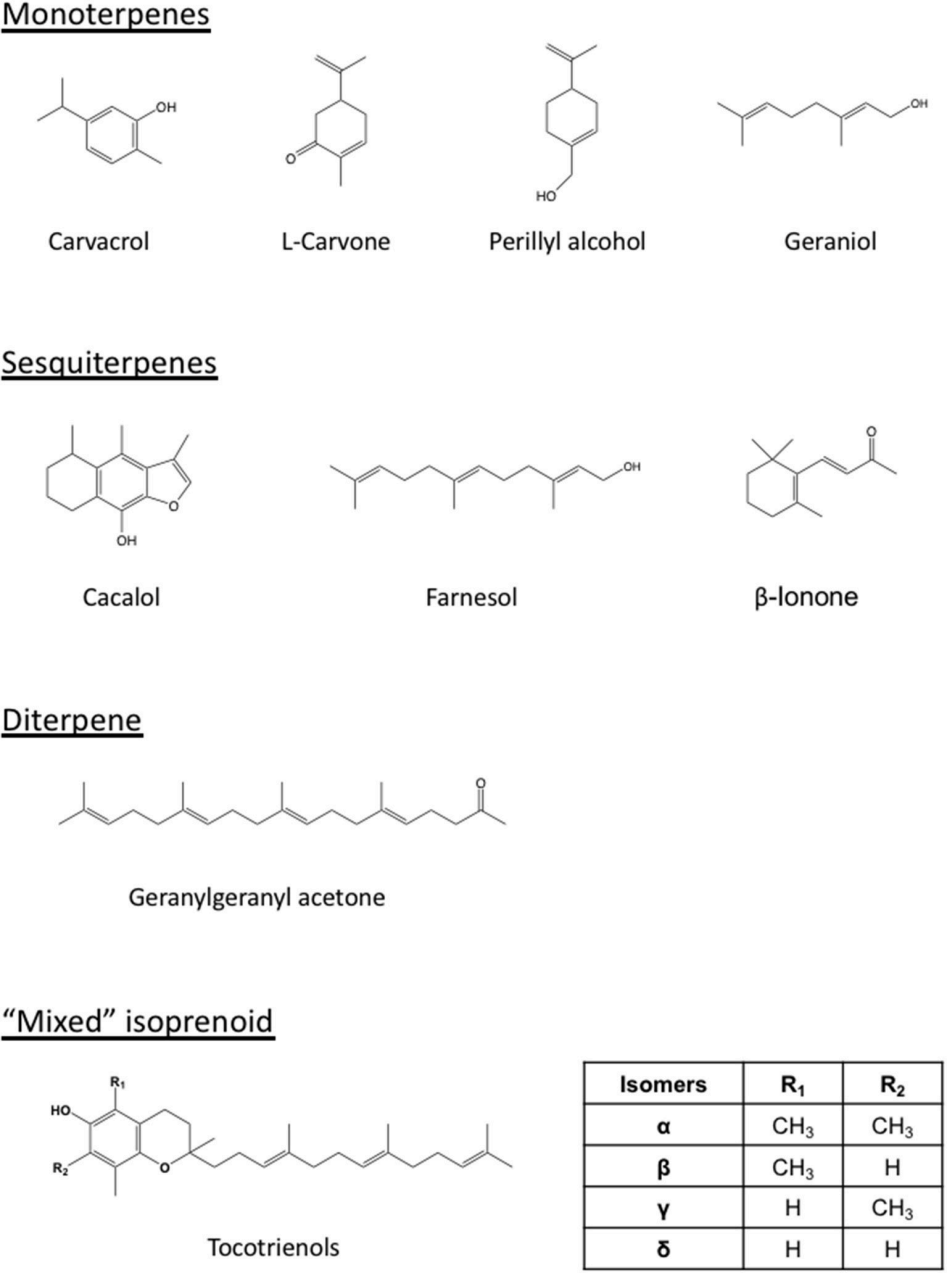
Structures of representative isoprenoids that impact tumors or potentiate the statin-mediated tumor suppression. Differing from the monoterpenes (carvacrol, carvone, perillyl alcohol, geraniol), sesquiterpenes (cacalol, farnesol, β-ionone), and diterpene (geranylgeranyl acetone), the tocotrienols are “mixed” isoprenoids with only part of their structure derived from the mevalonate pathway. The number and location of methyl group (R_1_ and R_2_) on the chromanol ring vary among the members (α, β, γ, and δ) of tocotrienols.

Isoprenoids have also shown synergistic effect with statins (Table 3). Figure 2 lists the structures of representative isoprenoids that potentiate the anti-cancer efficacy of statins *in vitro* and *in vivo*. All three two-way blends of tocotrienols, lovastatin, and β-ionone synergistically suppressed the proliferation of murine B16 melanoma and human A549 lung carcinoma, A2058 melanoma, MIA PaCa-2 pancreatic carcinoma, and DU145 prostate carcinoma cells (He et al., 1997; Mo and Elson, 1999; McAnally et al., 2007; Fernandes et al., 2010; Katuru et al., 2011). In human MCF-7 mammary tumor cells resistant to doxorubicin and tamoxifen, simvastatin and γ-tocotrienol synergistically eliminated cancer stem-like cells and reduced mammosphere formation and pStat-3 signaling; mevalonate supplementation attenuated the effect of the blend (Gopalan et al., 2013), suggesting that mevalonate depletion mediates the effect. Treatment of HepG2 hepatoma cells with a combination of 5 μM simvastatin and 50 μM geraniol, doses that cannot inhibit proliferation individually, significantly trapped tumor proliferation and inhibited cholesterol biosynthesis (Polo et al., 2011). Co-administration of pravastatin, a HMGCR inhibitor, and *d*-limonene, an inhibitor of protein prenylation, inhibited the growth of human HepG2 hepatoma cells by blocking post-translational modulation of p21^ras^ rather than suppression of cholesterol and dolichol biosynthesis (Kawata et al., 1994). An additive effect on cell growth and protein prenylation was shown with *d*-limonene and lovastatin in murine CT26 colon tumor cells (Broitman et al., 1996). Farnesyl-O-acetylhydroquinone, a farnesyl derivative, also synergized with lovastatin to suppress the proliferation and cell cycle progression of B16 melanoma cells (McAnally et al., 2003). Blends of statins and tocotrienols synergistically impacted an array of signaling molecules regulating cell cycle, cell proliferation, and apoptosis, including cyclin D1, CDK2, p27, MAPK, ERK, AKT, c-Jun N-terminal kinase (JNK), Rab, Rap1A, RhoA, caspase-3, and Ki-67, in human MCF-7 and MDA-MB-231 mammary tumor cells, + SA highly malignant mammary epithelial cells (Wali and Sylvester, 2007; Wali et al., 2009a,b; Ali et al., 2010; Alayoubi et al., 2012), MSTO, H2452, H2052, and H28 malignant mesothelioma cells (Tuerdi et al., 2013), and HT29 and HCT116 colon cancer cells (Yang et al., 2010). Blends of dietary lovastatin and *d*-δ-tocotrienol, but not individual agents, reduced the growth of implanted B16 melanoma in C57BL6 mice (McAnally et al., 2007).

**Table 3:** *In vitro* and *in vivo* studies showing that isoprenoids potentiate the tumor-suppressive effect of statins.

**Cell lines**	**Compounds**	**Impacts**	**References**
Human MCF-7 and MDA-MB-231 mammary tumor cells; +SA highly malignant mammary epithelial cells	Statins and γ-tocotrienol/tocotrienol-rich fraction in lipid nanoemulsion	Synergistic effect on cell viability, cell cycle arrest at G1 phase, ↑p27, ↑Rap1A; ↓cyclin D1, ↓CDK2, ↓pRb, ↓Ki-67, ↓p-p44 MAPK, ↓p-p38, ↓p-p54 JNK, ↓p-p46, ↓p-Akt, ↓Rab6, ↓p-ERK, ↓HMGCR; some of the effects attenuated by mevalonate	Wali and Sylvester, 2007; Wali et al., 2009a,b; Ali et al., 2010; Alayoubi et al., 2012
Human MCF-7 mammary tumor cells resistant to doxorubicin and tamoxifen	Simvastatin and γ-tocotrienol	Synergistic effect on eliminating cancer stem-like cells, ↓mammosphere formation and pStat-3 signaling; mevalonate attenuated the effect of the blend	Gopalan et al., 2013
Human HT29 and HCT116 colon cancer cells	Atorvastatin and γ-tocotrienol	Synergistic effect on cell proliferation and membrane RhoA; effects were attenuated by mevalonate; tocotrienol attenuated statin-induced upregulation of HMGCR	Yang et al., 2010
Human HepG2 hepatoma cells	Simvastatin and geraniol; pravastatin and *d*-limonene	Synergistic effect on Ras prenylation, DNA synthesis, cell proliferation and free and esterified cholesterol	Kawata et al., 1994; Polo et al., 2011
Human MSTO, H2452, H2052, and H28 malignant mesothelioma cells	Atorvastatin/simvastatin and γ-tocotrienol	Synergistic effect on cell viability (attenuated by GGPP and mevalonate), cell cycle, ↓HMGCR, ↓p-ERK/ERK; ↑caspase-3	Tuerdi et al., 2013
Human DU145 prostate carcinoma cells	Lovastatin and γ-tocotrienol/β-ionone	Synergistic effect on cell growth	Mo and Elson, 2004; Jones et al., 2013
Human MIA PaCa-2 pancreatic carcinoma cells	Lovastatin and δ-tocotrienol	Synergistic effect on cell growth	Hussein and Mo, 2009
Human A2058 melanoma cells	Lovastatin and δ-tocotrienol	Synergistic effect on cell growth	Fernandes et al., 2010
Murine B16 melanoma cells	Lovastatin and γ-tocotrienol/β-ionone /farnesyl-O-acetylhydroquinone	Synergistic effect on cell growth; additive effect on cell cycle progression	Mo and Elson, 1999; McAnally et al., 2003
Murine CT26 colon tumor cells	Lovastatin and perillyl alcohol	Additive effect on cell growth and protein prenylation	Broitman et al., 1996
**Animal Model**	**Compounds**	**Impacts**	**References**
C57BL6 mice	Dietary lovastatin and δ-tocotrienol	Blend of dietary lovastatin and δ-tocotrienol, but not individual agents, reduced the growth of implanted B16 melanoma	McAnally et al., 2007

The effect of tocotrienols on cell viability correlates with that on SREBP-2 activity, consistent with the finding that downregulation of SREBP2 and subsequent lipid biosynthesis effectively suppresses the growth of colon cancer proliferation and tumor spheroid formation (Wen et al., 2018). SREBP-2 and HMGCR were overexpressed in cisplatin-resistant A2780 epithelial ovarian cancer cells (Zheng et al., 2018). SREBP-2 promotes the expression of Staphylococcal nuclease and tudor domain containing 1 gene that is associated with the progression and malignancy of colon, breast, prostate, lung, glioma, skin, and liver cancers (Armengol et al., 2017), providing potential new applications for tocotrienols in cisplatin-resistant cancers. Mevalonate rescues the effect of statin, but not that of tocotrienol, on prostate cancer cell viability, consistent with tocotrienol-mediated increase in the degradation of nuclear, mature SREBP-2, which maintains cholesterol homeostasis through transcriptional regulation of enzymes in cholesterol biosynthesis and uptake beyond HMGCR (Krycer et al., 2012). The tocotrienol-enhanced degradation of SREBP-2 suggests that tocotrienol may impact, in addition to HMGCR, multiple enzymes of the mevalonate pathway, offering another aspect of synergy with statins, which specifically inhibit HMGCR. Such suitable and optimal combinations of statins and tocotrienol could lead to favorable synergistic effects with lower effective doses of statins with minimized or no side effects. The pair's combined lipid-lowering effects have been shown in chickens (Qureshi and Peterson, 2001) and hypercholesterolemic humans (Qureshi et al., 2001).

## Conclusion and Future Directions

The widely prescribed statins, originally designed for hypercholesterolemia, hold promises for cancer therapy as the multiple roles of the mevalonate pathway in growth support and regulation unfold. The repurposing of this class of well-established drugs for cancer stems from their ability to deplete mevalonate-derived sterol and nonsterol products essential for the prenylation of proteins involved in growth regulation and signaling pathways for cell proliferation. Nevertheless, the indiscriminate inhibition of HMGCR in normal and tumor cells and compensatory upregulation of HMGCR induced by statins may have contributed to their dose-limiting toxicities and conflicting clinical outcomes. The upregulated and sterol-resistant tumor HMGCR remains responsive to the isoprenoid-mediated downregulation, offering isoprenoids tumor-targeted growth suppression with little toxicity to normal cells. Isoprenoids also attenuate statin-induced compensatory upregulation of HMGCR via augmented, proteasome-mediated degradation of HMGCR, providing a new approach in enhancing the efficacy of statins with reduced adverse effects associated with lower statin doses. Most studies on the combination of isoprenoids and statins, however, are limited to *in vitro* models. A major gap hence remains in the lack of clinical data on the bioavailability, pharmacokinetics, pharmacodynamics, efficacy and mechanisms of action of the isoprenoids. The activities of the vast majority of the estimated 22,000 isoprenoids (Bach, 1995) remain unexplored. New formulation including those employing nanoparticles may improve the bioavailability of isoprenoids. Isoprenoid derivatives (Jung et al., 1998; Mo et al., 2000) with improved potencies and lower toxicities may provide more opportunities for optimal formulations. The combinations of isoprenoids and statins with complementary mechanisms and synergistic actions hold promise for cancer prevention and therapy and warrant further clinical studies.

## Author Contributions

HM, C-LS, and HY conceptualized the review. HM, RJ, AB, SY, and HY performed literature search. HM and SY constructed the figures. All authors participated in the writing and final editing of the manuscript.

### Conflict of Interest Statement

The authors declare that the research was conducted in the absence of any commercial or financial relationships that could be construed as a potential conflict of interest.

## Abbreviations

AMPK: adenosine monophosphate-activated protein kinase
CC-RCC: clear cell renal cell carcinoma
CDK2: cyclin dependent kinase 2
CHIP: Hsc70-interacting protein
DMBA, 7: 12-Dimethylbenz[a]anthracene
DNAJA1: DNAJ heat shock protein family (Hsp40) member A1
ERK: extracellular signal-regulated kinase
FPP: farnesol pyrophosphate
GGA: geranylgeranyl acetone
GGPP: geranylgeranyl pyrophosphate
Gp78: glycoprotein 78
HIF1α: hypoxia inducible factor 1α
HMG CoA: 3-hydroxy-3-methylglutaryl coenzyme A
HMGCR: 3-hydroxy-3-methylglutaryl coenzyme A reductase
HUVEC: umbilical vein endothelial cell
Insig: insulin-induced gene
JAK2: Janus Kinase 2
JNK: c-Jun N-terminal kinase
LDL: low density lipoprotein
MAPK: mitogen-activated protein kinase
MEP/DOXP: 2-C-methyl-D-erythritol 4-phosphate/1-deoxy-D-xylulose 5-phosphate
MNU: N-methyl-N-nitrosourea
mTOR: mammalian target of rapamycin
NFκB: nuclear factor kappa B
PI3K, phosphatidylinositol-4: 5-bisphosphate 3-kinase
SCAP: sterol regulatory element binding proteins cleavage protein
SRE: sterol regulatory element
SREBPs: sterol regulatory element binding proteins
STAT3: Signal transducer and activator of transcription 3
STOMP: Effect of Statins on Skeletal Muscle Function and Performance
TAZ: PDZ binding motif
TNM, Tumor, Node: Metastasis
TRF: tocotrienol-rich fraction
Ubc7: Ubiquitin-conjugating enzyme E2 7
UBIAD1: UbiA prenyltransferase domain-containing-protein-1
Ubiquinone: coenzyme Q_10_
VCP: valosin containing protein
VHL: Von Hippel-Lindau
YAP: yes-associated protein.
